# *Leishmania amazonensis*-Induced cAMP Triggered by Adenosine A_2B_ Receptor Is Important to Inhibit Dendritic Cell Activation and Evade Immune Response in Infected Mice

**DOI:** 10.3389/fimmu.2017.00849

**Published:** 2017-07-25

**Authors:** Amanda Braga Figueiredo, Míriam Conceição Souza-Testasicca, Tiago Wilson Patriarca Mineo, Luís Carlos Crocco Afonso

**Affiliations:** ^1^Laboratório de Imunoparasitologia, ICEB/NUPEB, Universidade Federal de Ouro Preto, Ouro Preto, Brazil; ^2^Coordenadoria da Área de Ciências Biológicas, Instituto Federal de Minas Gerais, Campus Ouro Preto, Ouro Preto, Brazil; ^3^Laboratório de Imunoparasitologia “Dr. Mario Endsfeldz Camargo”, ICBIM, Universidade Federal de Uberlândia, Uberlândia, Brazil

**Keywords:** A_2B_ adenosine receptor, cAMP, extracellular signal-regulated protein kinases, CD40, IL-12p70, dendritic cell, *Leishmania amazonensis*

## Abstract

Differently from others *Leishmania* species, infection by the protozoan parasite *L. amazonensis* is associated with a lack of antigen-specific T-cell responses. Dendritic cells (DC) are essential for the innate immune response and for directing the differentiation of T-helper lymphocytes. Previously, we showed that *L. amazonensis* infection impairs DC activation through the activation of adenosine A_2B_ receptor, and here, we evaluated the intracellular events triggered by this receptor in infected cells. To this aim, bone marrow-derived DC from C57BL/6J mice were infected with metacyclic promastigotes of *L. amazonensis*. Our results show, for the first time, that *L. amazonensis* increases the production of cAMP and the phosphorylation of extracellular signal-regulated protein kinases 1/2 (ERK1/2) in infected DC by a mechanism dependent on the A_2B_ receptor. Furthermore, *L. amazonensis* impairs CD40 expression and IL-12 production by DC, and the inhibition of adenylate cyclase, phosphoinositide 3-kinase (PI3K), and ERK1/2 prevent these effects. The increase of ERK1/2 phosphorylation and the inhibition of DC activation by *L. amazonensis* are independent of protein kinase A (PKA). In addition, C57BL/6J mice were inoculated in the ears with metacyclic promastigotes, in the presence of PSB1115, an A_2B_ receptor antagonist. PSB1115 treatment increases the percentage of CD40^+^ DC on ears and draining lymph nodes. Furthermore, this treatment reduces lesion size and tissue parasitism. Lymph node cells from treated mice produce higher levels of IFN-γ than control mice, without altering the production of IL-10. In conclusion, we suggest a new pathway used by the parasite (A_2B_ receptor → cAMP → PI3K → ERK1/2) to suppress DC activation, which may contribute to the decrease of IFN-γ production following by the deficiency in immune response characteristic of *L. amazonensis* infection.

## Introduction

*Leishmania* parasites are protozoa transmitted between their hosts by female sand flies and cause in humans a group of diseases known as leishmaniasis. These diseases present a wide spectrum of clinical manifestations dependent on the parasite species and the host immune response. *Leishmania amazonensis* (*L. amazonensis*), in humans, causes diffuse leishmaniasis that is associated with diffuse non-ulcerative lesions with innumerous parasites. In this case, the parasite resistance to treatment is common (1, 2). The deficiency in immune responses associated with *L. amazonensis* infection, characterized by a lack of antigen-specific T-cell responses, contributes significantly to the failure of therapeutic approaches. Therefore, the understanding of evasion mechanisms used by *L. amazonensis* during infection has much to contribute to the development of new therapeutic strategies. In the murine model, this parasite causes non-healing chronic lesions in mouse strains otherwise resistant to other *Leishmania* species, such as *Leishmania braziliensis* and *Leishmania major* (3–5). The murine model has been extensively used to evaluate the mechanisms involved in the activation/evasion of the host immune response by the parasite.

Dendritic cells (DC) are essential players in the fight against infection where they link the innate and acquired immune responses. The role of these cells in infections induced by *Leishmania* has been clearly demonstrated. After contact with microorganisms, these cells initiate a maturation process characterized by increased expression of MHC class II and co-stimulators, such as CD80, CD86, and CD40 (6). Importantly, CD40–CD40L interaction is essential for antigen-specific T-helper lymphocyte priming (7, 8). Additionally, DC produce a wide array of cytokines and can direct T-helper cell differentiation (9). In this way, IL-12 production by DC induces the differentiation of IFN-γ-producing Th1 lymphocytes, which are critical to the control of *Leishmania* replication in the infected host (10–12). Several studies evaluated the interaction between *Leishmania* parasites and DC demonstrating that *L. amazonensis* can modulate several DC functions by modifying the expression of MHC class II, CD80 and CD86 and the production of IL-10 and IL-12 (13–18).

One important aspect of the infection by *L. amazonensis* in the murine model is the fact that, contrary to other *Leishmania* species, no mouse strain is completely resistant to the parasite [reviewed by Pereira and Alves (19)]. In addition, with the exception of BALB/c mice, the susceptibility to *L. amazonensis* infection is independent of disease-inducing cytokines such as IL-4 or IL-10, regardless of number or stage of development (purified metacyclic or stationary phase) of the promastigotes used for infection as well as the site of the infection (20–22). IL-10 only seems to play a relevant role, when its production is increased at the site of infection by the administration of sandfly saliva or adenosine and AMP (22, 23). Thus, finding an alternative immunomodulatory mechanism distinct from the participation of regulatory cytokines has been the aim of our laboratory for the last 15 years.

Extracellular ATP, released during infection or cellular injury, acts as a danger signal and a potent stimulator of inflammatory responses (24–26). Ectonucleotidases CD39 and CD73 hydrolyze ATP to adenosine, the latter of which presents immunomodulatory properties, such as inhibition of the production of inflammatory cytokines, such as TNF-α and IL-12, and stimulating the production of IL-10 (27, 28). Adenosine can act through A_1_, A_2A_, A_2B_, and A_3_ receptors. A_2_ receptors are able to stimulate adenylate cyclase, leading to the accumulation of cAMP (29–31), which impairs CD40 expression, the generation of inflammatory mediators, IL-12 production and microbicidal activity (32, 33).

Previously, we showed that *L. amazonensis* infection impairs DC activation (by decreasing the expression of MHC class II, CD86, and CD40) and, as a consequence, the triggering of an antigen-specific cellular response. This effect was dependent on the activation of A_2B_ receptor (34), but the signaling pathways activated by this receptor remained unknown. Similarly, other studies demonstrated that inhibition of *L. amazonensis*-stimulated extracellular signal-regulated protein kinases 1/2 (ERK1/2) phosphorylation increases CD40 expression on DC (35) and decreases lesion size in mice infected by this parasite (36).

Given that *L. amazonensis* decreases DC activation, in particular CD40 expression, *via* A_2B_ receptor, in this study, we evaluated the intracellular events triggered by this receptor in infected cells. Furthermore, we evaluated the role of A_2B_ receptor on lesion development in mice infected by *L. amazonensis*. Our results show that *L. amazonensis* increases cAMP production by DC and stimulates the phosphorylation of ERK1/2 in these cells by mechanisms dependent on A_2B_ receptor. Adenylate cyclase, phosphoinositide 3-kinase (PI3K), and ERK1/2 are involved in the decreased CD40 expression and IL-12 production in *L. amazonensis*-infected DC. In addition, A_2B_ receptor blockade controls lesion development in mice infected by *L. amazonensis*, probably by increasing the percentage of CD40^+^ DC and the production of IFN-γ by lymph node cells.

## Materials and Methods

### Animals and Parasites

C57BL/6J (2–6 months old) mice were obtained from the Universidade Federal de Ouro Preto animal facility. Animals received water and food *ad libitum*. This study was carried out in accordance with the recommendations of the Brazilian Guidelines for animal experimentation. The protocols were approved by the University’s Ethical Committee on Animal Experimentation (CEUA 2012/02 and CEUA 2013/51). *Leishmania amazonensis*, PH8 strain (IFLA/BR/67/PH8) promastigotes were grown in Grace’s medium (Sigma-Aldrich, St. Louis, MO, USA) supplemented with 10% heat-inactivated fetal calf serum (FCS, Cultilab, Campinas, SP, Brazil), 2 mM l-glutamine (Sigma-Aldrich) and 100 U/mL penicillin G potassium (Sigma-Aldrich), pH 6.5, at 25°C. Metacyclic promastigotes were purified by gradient centrifugation of parasites at the stationary phase of culture (day 5) over Ficoll 400 (Sigma-Aldrich), as previously described (5). In *in vitro* DC infection experiments, metacyclic promastigotes, suspended in PBS with 5% FCS, were incubated in the presence of 5 µM CFSE (Sigma-Aldrich) at 37°C for 10 min in the dark. The suspension was centrifuged and the parasites were washed in PBS, pH 7.2 (37). Alternatively, parasites were labeled with PKH26 (Sigma-Aldrich) according to the manufacturer’s instructions.

### Differentiation of Bone Marrow-Derived DC

Bone-marrow-derived DC were obtained from C57BL/6J bone marrow as previously described (38). Briefly, bone marrow cells were isolated from the femur and tibia of C57BL/6J mice. Bone marrow cell suspensions were centrifuged and cells cultured in RPMI-1640 (Sigma-Aldrich) supplemented with 10% FCS, 2 mM l-glutamine, 100 U/mL penicillin G potassium, and 50 µM β-mercaptoetanol (Pharmacia Biotech AB, Uppsala, Sweden), pH 7.2. Cells were plated in Petri dishes at a concentration of 3 × 10^5^ cells/mL and incubated at 37°C/5% CO_2_. GM-CSF (R&D Systems, Minneapolis, MN, USA) was added to each plate on the days 0, 3, and 6, at a concentration of 3 ng/mL (1,050 U/mL). Non-adherent DC were collected on the ninth day of culture. In regard to a recently published work (39), DC were extensively characterized. DC were CD11b^+^CD11c^+^F4/80^−/low^MHCII^+^ cells and showed morphology characteristic of this population, with several and irregular dendrites. In addition, these cells were able to stimulate mixed leukocyte reaction and antigen-specific proliferation of CD4^+^ T lymphocyte (data not shown).

### *In Vitro* DC Infection

CFSE-labeled metacyclic promastigotes and DC were co-incubated (1:3 cell to parasite ratio) in RPMI-1640 supplemented with 10% FCS, 2 mM l-glutamine, 100 U/mL penicillin G potassium, and 50 µM β-mercaptoetanol (Pharmacia Biotech AB, Uppsala, Sweden), pH 7.2, at 33°C/5% CO_2_ for 3 h and subsequently incubated at 37°C/5% CO_2_ for up to 17 h. In selected experiments A_2B_ adenosine receptors antagonist, MRS1754 {*N*-(4-cyanophenyl)-2-[4-(2,3,6,7-tetrahydro-2,6-dioxo-1,3-dipropyl-1*H*-purin-8-yl)phenoxy]-acetamide, Tocris Bioscience, Park Ellisville, MO, USA}, or inhibitors of adenylate cyclase [SQ22536, 9-(tetrahydro-2-furanyl)-9*H*-purin-6-amine, Tocris Bioscience], protein kinase A (PKA) (KT5720, (9*R*,10*S*,12*S*)-2,3,9,10,11,12-hexahydro-10-hydroxy-9-methyl-1-oxo-9,12-epoxy-1*H*-diindolo[1,2,3-*fg*:3’,2’,1’-*kl*]pyrrolo[3,4-*i*][1,6]benzodiazocine-10-carboxylic acid, hexyl ester, Sigma-Aldrich), PI3K [LY294002, 2-(4-morpholinyl)-8-phenyl-4*H*-1-benzopyran-4-one hydrochloride, Sigma-Aldrich] or ERK1/2 (U0126, 1,4-diamino-2,3-dicyano-1,4-*bis*[2-aminophenylthio]butadiene, Tocris Bioscience) were added at the moment of DC infection as described in figure legends. All drugs were diluted in DMSO (1% final concentration), which was added to control cultures.

### cAMP Measurement

Briefly, metacyclic promastigotes and DC were co-incubated as already described, in the presence of 0,1 mM Ro 20-1724 (phosphodiesterase inhibitor, Sigma-Aldrich), at 33°C/5% CO_2_ for 15 min. In selected groups, MRS1754 was added at the moment of DC infection. In other groups, DC previously co-incubated with parasites and adenosine receptor antagonists were stimulated with 1 µM 5′-(N-ethylcarboxamido) adenosine (NECA, Sigma-Aldrich) at 33°C/5% CO_2_ for more 15 min. cAMP was measured by a bioluminescent assay according to the manufacturer’s instructions (Promega, Madison, WI, USA).

### Infection of Mice

Female C57BL/6J mice were inoculated intradermally in the ears with 10^3^ or 10^5^ metacyclic promastigotes of *L. amazonensis*, in the presence or absence of 5 µM PSB1115 [1-propyl-8-(4-sulfophenyl) xanthine potassium salt hydrate]. Lesion size was measured weekly with a digital micrometer (Starrett, Athol, MA, USA). The lesion size was defined as the difference between the thickness of the infected and uninfected ears.

### Culture of Lymph Node Cells

Single-cell suspensions were prepared from the auricular lymph nodes of mice infected for 12 weeks. Cell concentration was adjusted to 5 × 10^6^ cells/mL in DMEM (Sigma-Aldrich) supplemented with 10% FCS, 2 mM l-glutamine, 100 U/mL penicillin G, 50 µM β-mercaptoethanol and 25 mM HEPES (Sigma-Aldrich), pH 7.2. Cell suspensions were distributed in culture plates and stimulated with 50 µg/mL of *L. amazonensis* particulate antigen. Supernatants were harvested after 48 h.

### Parasite Load Estimation

The number of parasites in the ear lesion was estimated by the limiting dilution assay (3). After 12 weeks of infection, mice were euthanized and the ears removed and incubated in RPMI-1640/1 mg/mL collagenase A, pH 7.2, for 2 h at 37°C/5% CO_2_. The ears were ground in Grace’s medium, pH 6.5. Tissue debris were removed by centrifugation. Cells were resuspended in Grace’s medium supplemented with 10% FCS, 2 mM l-glutamine and 100 U/mL penicillin G, pH 6.5. The parasite suspension was serially diluted in 10-fold dilutions, pipette tips were replaced for each dilution. After 2 weeks of incubation at 25°C, plates were examined under an inverted microscope for the presence of parasites. Results were expressed as −log of the number of parasites corresponding to the last dilution in which they were observed.

### Isolation of Cells from Ears and Draining Lymph Nodes

C57BL/6J mice were inoculated intradermally in both ears with 10^5^ metacyclic promastigotes of *L. amazonensis*, in the presence or absence of 5 µM PSB1115. 7 days after infection, both ears and draining auricular lymph nodes were removed. Only ears were incubated in RPMI-1640/1 mg/mL collagenase A, pH 7.2, for 2 h at 37°C/5% CO_2_. The ears were ground in RPMI-1640, pH 7.2, using a BD Medimachine™ system and the suspension was filtered through a 30 µm Filcon (BD Biosciences, San Jose, CA, USA). The lymph nodes were ground in RPMI-1640, pH 7.2, using a tissue homogeneizer. Cells were stained and analyzed by flow cytometry as described below.

### Cytokine Measurement

Supernatants from DC cultures were collected after 20 h and supernatants from lymph node cell cultures after 48 h and IL-12p70, IL-10, and IFN-γ cytokine levels were measured by ELISA using kits according to the manufacturer’s instructions (BD OptEIA, San Diego, CA, USA).

### Flow Cytometry

For surface markers staining, cells in PBS with 1% BSA were submitted to FcγR blocking in the presence of anti-mouse CD16/CD32 (produced in our laboratory). Subsequently, cells were incubated with anti-mouse CD11c (HL3 clone), anti-mouse CD40 (3/23 clone—BD Pharmingen, San Diego, CA, USA), or their respective isotype controls, at 4°C for 30 min in the dark. The suspensions were centrifuged and the cells were washed in PBS, pH 7.2 and resuspended in a solution of 1% paraformaldehyde, 47.7 mM sodium cacodylate, and 113 mM NaCl, pH 7.2. Intracellular phospho-protein staining was performed according to the manufacturer’s instructions (BD Phosflow, San Diego, CA, USA). Briefly, DC previously co-incubated with parasites were stimulated with 1 µM NECA at 33°C/5% CO_2_ for 15 min, fixed in Lyse/fix buffer, permeabilized with Perm buffer III and incubated with anti-ERK1/2 pT202/pY204 (20 A clone), or its respective isotype control, at room temperature for 60 min in the dark. The suspensions were centrifuged and the cells were washed in PBS, pH 7.2 and resuspended in Stain buffer. The samples were analyzed in BD FACSCalibur™ flow cytometer. Cell acquisition was performed using BD CellQuest™ Pro software. Data analysis was performed using FlowJo software.

### Statistical Analysis

Student’s *t*-test and one-way ANOVA were performed using Prism 5.0 software (GraphPad Software, La Jolla, CA, USA). *p* < 0.05 was considered statistically significant.

## Results

### *L. amazonensis* Infection Increases cAMP Production, Which Impairs DC Activation

As previously stated, our group showed that *L. amazonensis* impairs DC activation, especially CD40 expression, by a mechanism dependent on the A_2B_ receptor (34). Here, we decided to investigate the signaling pathways involved in this process. In the figures, a schematic of the possible pathways triggered by the A_2B_ receptor is shown, highlighting the pathways activated in *L. amazonensis*-infected DC.

**Figure 1:**
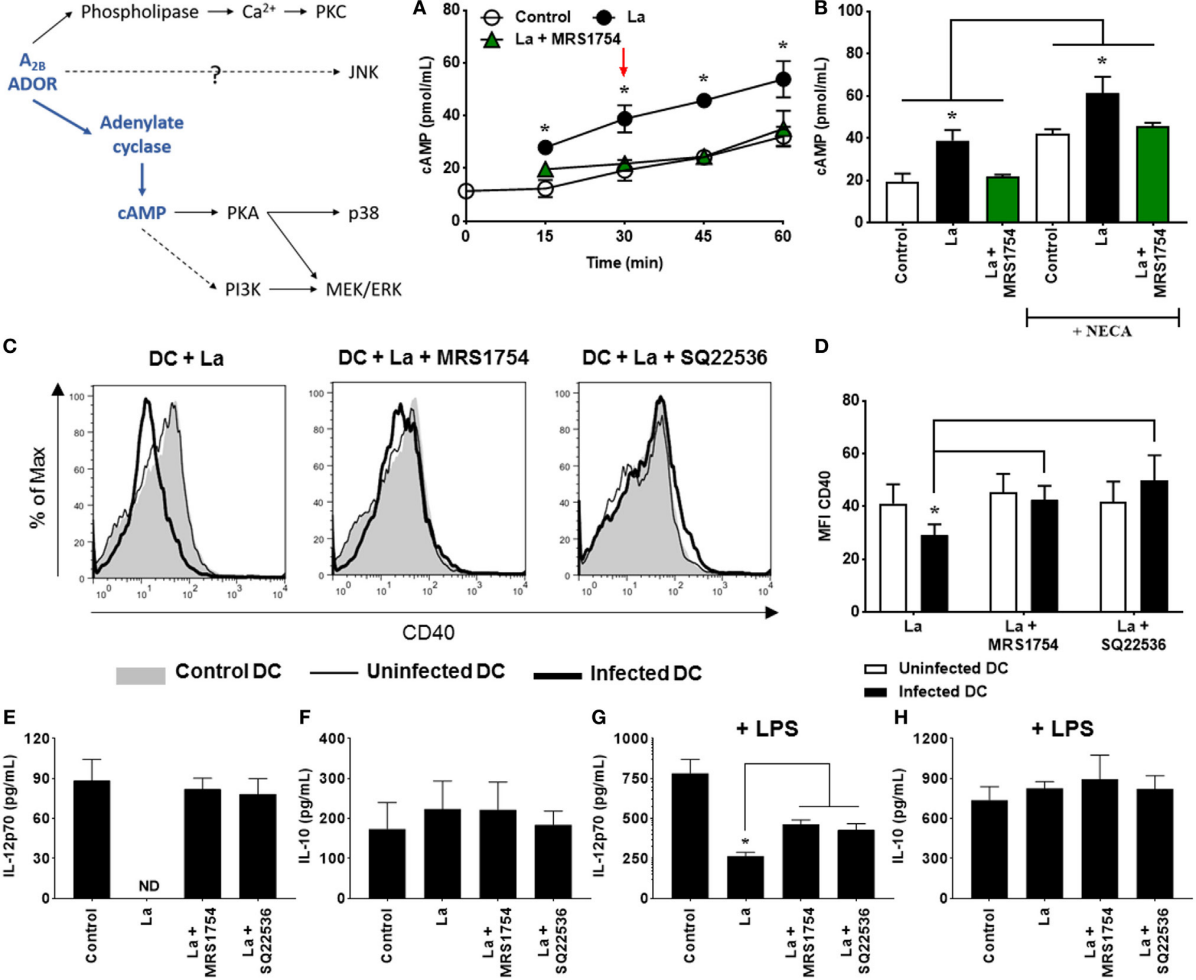
*Leishmania amazonensis* infection increases cAMP production by dendritic cells (DC), that is involved in the decrease of CD40 expression and IL-12p70 production. **(A)** DC obtained after 9 days of culture with GM-CSF were infected with metacyclic promastigotes (1:3 cell to parasite ratio) in the presence of 0.1 mM Ro 20-1724 and 5 µM MRS1754 (adenosine A_2B_ receptor antagonist) and the cAMP production evaluated after 15–60 min. **(B)** After 30 min of infection (as shown by arrow in graph A), DC were stimulated with 1 µM NECA (non-selective adenosine receptor agonist) for more 15 min. The results represent the mean + SD from three independent experiments. *p < 0.05 between La and Control or La + MRS1754 groups, or between linked groups, two-tailed Student’s *t*-test. **(C–H)** DC were infected with CFSE-labeled metacyclic promastigotes in the presence of 5 µM MRS1754 or 100 µM SQ22536 (adenylate cyclase inhibitor) and CD40 expression and IL-12p70 and IL-10 production evaluated after 20 h. **(C,D)** CD11c^+^ DC were gated into populations of uninfected (CFSE^−^ cells, white bars) and infected (CFSE^+^ cells, black bars) cells and the MFI of CD40 analyzed in both populations. Control is DC that had not contact with parasites. **(C)** Histograms are representative of at least three independent experiments. IL-12p70 **(E,G)** and IL-10 **(F,H)** cytokine levels were measured in the supernatants using an ELISA. **(G,H)** 2 µg/mL LPS was added after 3 h of infection. ND, not detected. The results represent the mean + SD from five independent experiments. *p < 0.05 between uninfected and infected DC or between linked groups, two-way ANOVA and Tukey’s post-test **(A,B,D)** two-tailed Student’s *t*-test **(G)**.

Adenosine A_2B_ receptors are G protein-coupled receptors that can be associated both to α_s_, leading to cAMP production, or to α_q_ subunits, which stimulate phospholipase C and accumulation of intracellular calcium (29). Thus, to evaluate the accumulation of cAMP, DC were infected with *L. amazonensis*, in the presence or absence of MRS1754, a selective A_2B_ receptor antagonist, and cAMP levels measured by a chemiluminescence assay before and after the addition of NECA, a non-selective adenosine receptor agonist. As shown in Figures 1A,B, *L. amazonensis* infection significantly increases cAMP production by DC. Interestingly, the blockade of A_2B_ receptor reverses this effect. Addition of NECA to the culture does not substantially increase cAMP levels. The fact that cAMP production was inhibited by MRS1754 even in the absence of an exogenous stimulus (NECA) suggests that some level of extracellular adenosine production is present during the interaction between the parasite and the host cell.

Previous studies have shown that cAMP plays a critical role in the inhibition of immune cells (32), including DC (40, 41). To confirm that cAMP production induced by A_2B_ receptor activation is important for the inhibition of DC activation by *L. amazonensis*, we evaluated the expression of CD40 and the production of IL-12p70 and IL-10 in cells treated with SQ22536, an inhibitor of adenylate cyclase. As previously shown (34), *L. amazonensis* inhibits CD40 expression on DC and this effect is abolished in the presence of MRS1754 (Figures 1C,D). In addition, we showed that inhibition of adenylate cyclase by SQ22536 treatment also restores CD40 expression on infected DC (Figures 1C,D). Moreover, cells infected with *L. amazonensis* are unable to produce basal levels of IL-12p70, but this ability is restored after the blockade of A_2B_ receptor or inhibition of adenylate cyclase (Figure 1E). The same effect is observed when we stimulated infected cells with LPS (Figure 1G). Finally, we find no changes in IL-10 production by infected DC as compared to uninfected DC and MRS1754 or SQ22536 treatments do not interfere with IL-10 production by these cells (Figures 1F,H). Taken together, our first set of results show that *L. amazonensis* infection increases cAMP production by DC, and that the production of this intracellular messenger is critical for the decrease of CD40 expression and IL-12p70 production by infected cells.

In addition to Gs proteins, the adenosine A_2B_ receptor has been shown to also engage Gq protein capable of stimulating intracellular calcium accumulation (29). To exclude the role of calcium in DC inhibition by *L. amazonensis, L. amazonensis* metacyclic promastigotes were labeled with PKH26 and used to infected DC cells loaded with Oregon Green 488. Our results show that although *L. amazonensis* infection increases the amount of intracellular calcium in DC, this increase is independent of A_2B_ receptor activation, since treatment with MRS1754 does not reverse calcium accumulation (Figure S1A in Supplementary Material). Furthermore, we observe no change in intracellular calcium levels after addition of NECA, a non-selective adenosine receptor agonist, both in uninfected cells and infected cells (Figure S1A in Supplementary Material). These results show that *L. amazonensis* infection leads to the accumulation of calcium in DC, but adenosine is not responsible for this effect. Moreover, since calcium accumulation triggers protein kinase C (PKC) activation, DC were infected in the presence of staurosporine, a PKC inhibitor, and this treatment was unable to reverse the inhibition of CD40 expression and IL-12p70 production in infected cells (Figures S1B–D in Supplementary Material) confirming that calcium accumulation was not related to inhibition of CD40 expression.

### Decrease of CD40 Expression and IL-12p70 Production by *L. amazonensis*-Infected DC Is Dependent on PI3K

**Figure 2:**
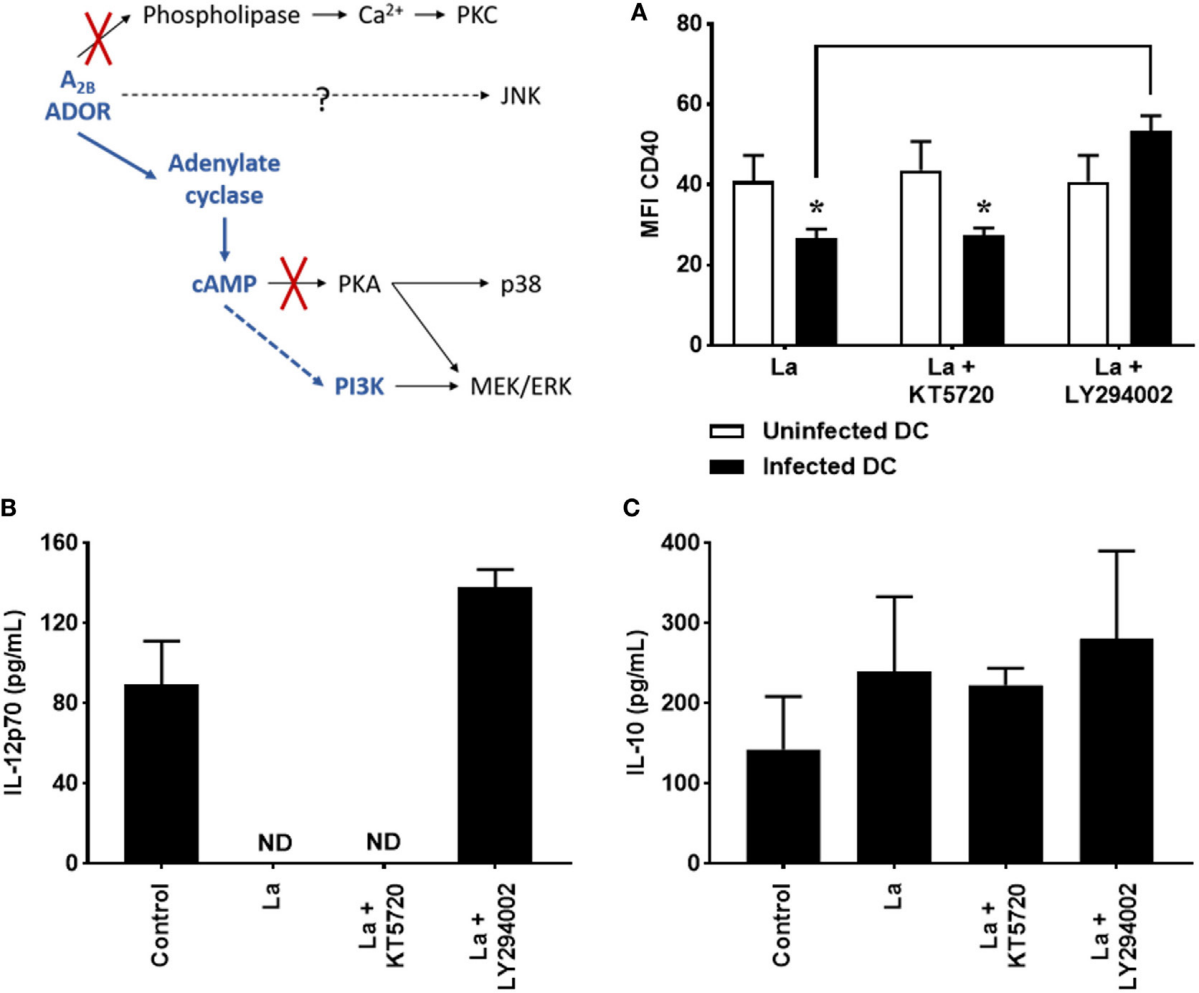
Decrease of CD40 expression and IL-12p70 production by *Leishmania amazonensis*-infected dendritic cell (DC) is dependent on phosphoinositide 3-kinase (PI3K). DC were infected as described in Figure 1 in the presence of 5 µM MRS1754 (adenosine A_2B_ receptor antagonist) or 10 µM KT5720 [protein kinase A (PKA) inhibitor] or 1 µM LY294002 (PI3K inhibitor) and CD40 expression and IL-12p70 and IL-10 production evaluated after 20 h. **(A)** CD11c^+^ DC were gated into populations of uninfected (CFSE^−^ cells, white bars) and infected (CFSE^+^ cells, black bars) cells and the MFI of CD40 analyzed in both populations. IL-12p70 **(B)** and IL-10 **(C)** cytokine levels were measured in the supernatants using an ELISA. Control is uninfected DC. ND, not detected. The results represent the mean + SD from three independent experiments. *p < 0.05 between uninfected and infected DC or between linked groups, two-way ANOVA and Tukey’s post-test.

cAMP can binds PKA or Epac (exchange protein activated by cyclic AMP), which is able to phosphorylate residues on several target proteins (32). cAMP may also lead to the activation of another kinase, PI3K (29, 42). In order to verify whether these proteins are involved in cAMP-mediated DC inhibition, cells were infected by *L. amazonensis* in the presence of KT5720 or LY294002, known inhibitors of PKA and PI3K, respectively, and after 20 h of infection, CD40 expression and cytokine production were evaluated. As shown in Figure 2, inhibition of PKA does not modify CD40 expression (Figure 2A) and IL-12p70 production (Figure 2B). In addition, it also does not alter IL-10 production (Figure 2C) by *L. amazonensis*-infected cells. On the other hand, treatment with LY294002 significantly increases CD40 expression and IL-12p70 production by infected cells, showing that PI3K activation takes part in the inhibition of DC activation by *L. amazonensis*.

### ERK1/2 Is Involved in the Decrease of CD40 Expression and IL-12p70 Production by *L. amazonensis*-Infected DC

Mitogen-activated protein kinases (MAPK) is a diverse protein family that consists of three main groups: the c-Jun N-terminal kinases (JNK), the stress-activated protein kinase (SAPK) p38, and the extracellular signal-regulated protein kinases (ERK). These kinases are involved in intracellular signaling events triggered by adenosine (29). Although *L. amazonensis* infection increased the phosphorylation of JNK and p38, these effects are independent of the A_2B_ receptor; furthermore, the inhibition of both proteins is unable to alter the levels of CD40 expression and the production of IL-12p70 and IL-10 by infected cells (Figure S2 in Supplementary Material).

**Figure 3:**
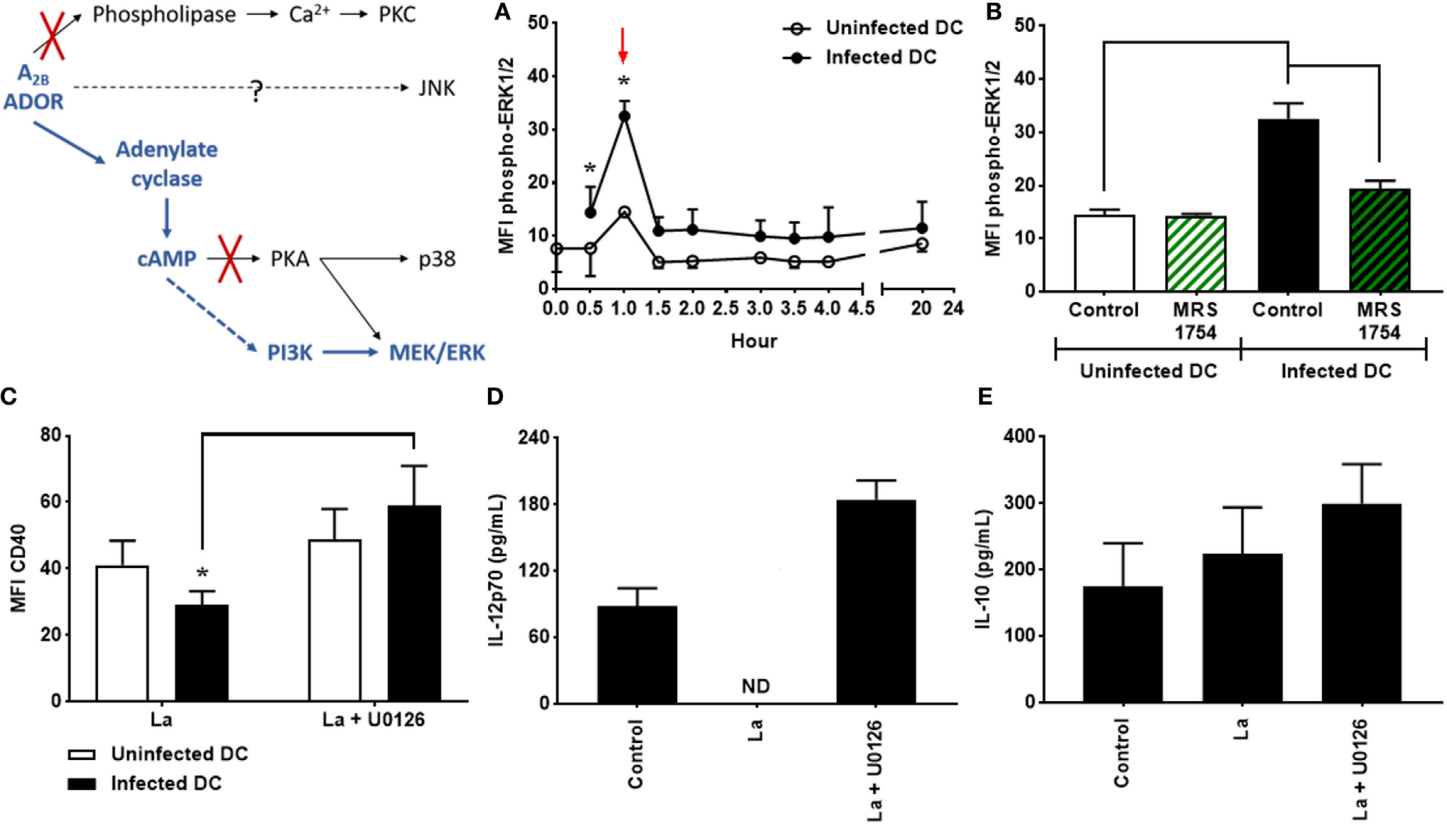
Extracellular signal-regulated protein kinases 1/2 (ERK1/2) is involved in the decrease of CD40 expression and IL-12p70 production by *Leishmania amazonensis*-infected dendritic cells (DC). **(A)** DC were infected as described in Figure 1 and the MFI of phospho-ERK1/2 analyzed in populations of uninfected and infected DC after multiple periods of infection. After infection, cells were stimulated with 1 µM NECA for 15 min, fixed and then analyzed by flow cytometry. **(B)** MFI of phospho-ERK1/2 in uninfected and infected CD11c^+^ DC in the absence (Control) or presence of 5 µM MRS1754 (adenosine A_2B_ receptor antagonist), after 1 h of infection (as shown by arrow in graph A). The results represent the mean + SD from three independent experiments. *p < 0.05 between uninfected and infected DC or between linked groups, two-way ANOVA and Tukey’s post-test. **(C–E)** DC were infected in the presence of 5 µM MRS1754 or 10 µM U0126 (MEK inhibitor) and CD40 expression and IL-12p70 and IL-10 production evaluated after 20 h. **(C)** CD11c^+^ DC were gated into populations of uninfected (CFSE^−^ cells, white bars) and infected (CFSE^+^ cells, black bars) cells and the MFI of CD40 analyzed in both populations. IL-12p70 **(D)** and IL-10 **(E)** cytokine levels were measured in the supernatants using an ELISA. Control is uninfected DC. ND, not detected. The results represent the mean + SD from five independent experiments. *p < 0.05 between uninfected and infected DC or between linked groups, two-way ANOVA and Tukey’s post-test.

As previously mentioned, it has been shown that *L. amazonensis* is able to stimulate the phosphorylation of ERK1/2, which is involved in the inhibition of CD40 on *L. amazonensis*-infected DC (35, 43); however, the mechanisms that lead to the activation of ERK1/2 in infected DC remain unknown. To investigate whether the activation of the A_2B_ receptor is involved in ERK1/2 phosphorylation, DC were infected with *L. amazonensis* in the presence or absence of MRS1754. As shown in Figure 3A, phosphorylation of ERK1/2 is significantly higher in infected cells if compared to uninfected controls, after 1 h of infection. Interestingly, the blockade of the A_2B_ receptor by MRS1754 treatment completely abrogates this effect (Figure 3B), demonstrating that the A_2B_ receptor is critical for the phosphorylation of ERK1/2 induced by *L. amazonensis* infection.

In addition, DC infection in the presence of U0126, a potent inhibitor of MEK, results in a considerable increase in the expression levels of CD40 in infected cells (Figure 3C). Furthermore, *L. amazonensis*-infected DC treated with U0126 recover their ability to produce IL-12p70 (Figure 3D). Again, IL-10 production by DC is not affected (Figure 3E). The same effects are observed in DC infected in the presence of PD98059, another inhibitor of ERK1/2 (data not shown). Taken together, our results show that ERK1/2 phosphorylation driven by A_2B_ receptor activation plays a relevant role in the inhibition of CD40 expression and IL-12p70 production by *L. amazonensis*-infected DC.

**Figure 4:**
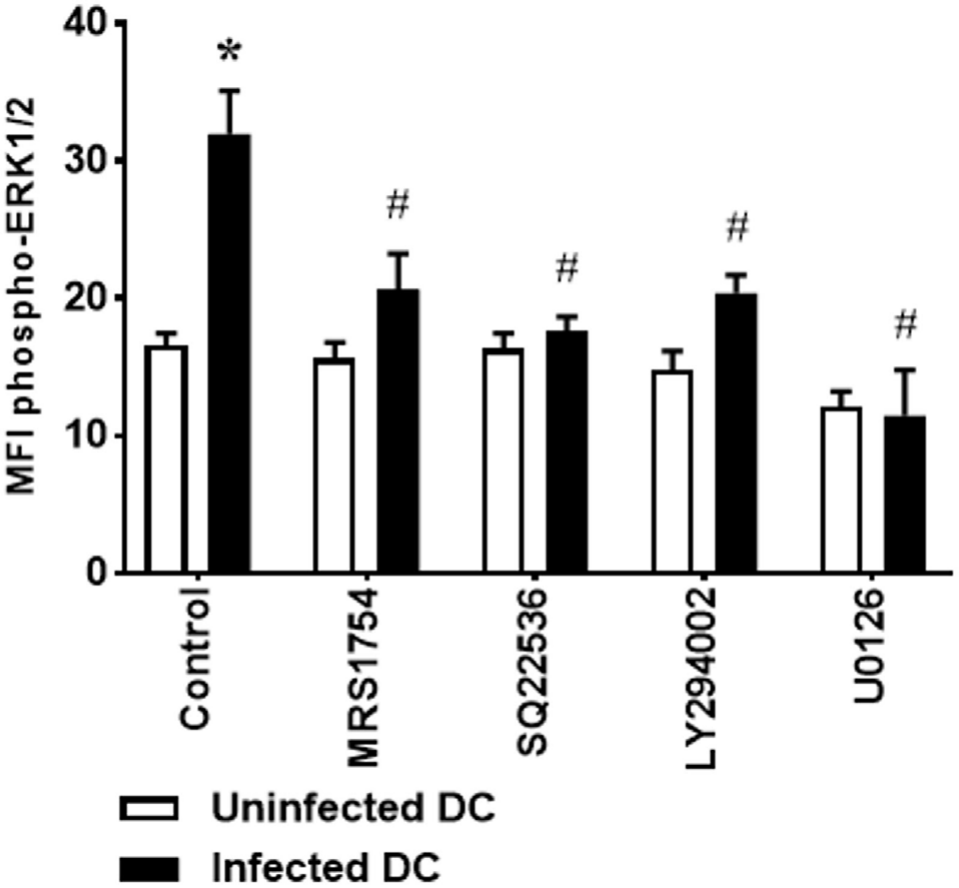
Extracellular signal-regulated protein kinases 1/2 (ERK1/2) phosphorylation in *Leishmania amazonensis*-infected dendritic cells (DC) is dependent on A_2B_ receptor, AMPc production, and phosphoinositide 3-kinase (PI3K) activation. DC were infected as described in Figure 1, in the absence (Control) or presence of 5 µM MRS1754 (adenosine A_2B_ receptor antagonist), 100 µM SQ22536 (adenylate cyclase inhibitor), 1 µM LY294002 (PI3K inhibitor), or 10 µM U0126 (MEK inhibitor), and the MFI of phospho-ERK1/2 analyzed in populations of uninfected and infected CD11c^+^ DC after 1 h of infection. After infection, cells were stimulated with 1 µM NECA for 15 min, fixed and then analyzed by flow cytometry. The results represent the mean + SD from three independent experiments. *p < 0.05 between uninfected and infected DC, ^#^*p* < 0,05 between Control and treated DC, two-way ANOVA and Tukey’s post-test.

Our results demonstrate that inhibition of CD40 expression and IL-12p70 production by *L. amazonensis*-infected DC is dependent on A_2B_ receptor activation, cAMP production, PI3K activation, and ERK1/2 phosphorylation. To assess whether ERK1/2 phosphorylation, as previously found for A_2B_ receptor, is also dependent on the cAMP production and PI3K activation, DC were infected in the presence of SQ22536 or LY294002. U0126 was used as control. Interestingly, both adenylate cyclase and PI3K inhibition are able to reverse the phosphorylation of ERK1/2 stimulated by *L. amazonensis* (Figure 4), suggesting that A_2B_ receptor, adenylate cyclase, PI3K, and ERK1/2 act in a direct pathway, instead of these proteins act in parallel and independent pathways.

In our previous study, we observed that infection by *L. amazonensis* in addition to decrease CD40 expression, also inhibited the expression of MHCII and CD86. We also demonstrated that the inhibition of the expression of these molecules was reversed by the A_2B_ receptor antagonist, MRS1754. To verify if the pathway involved in the inhibition of CD40 expression is also associated with the inhibition of MHCII and CD86 expression, we analyzed the expression of these molecules in the presence of the inhibitors of the key enzymes studied here. As shown in Figure S3 in Supplementary Material, although the blockade of the A_2B_ receptor is able to reverse the inhibition of MHCII and CD86, inhibition of adenylate cyclase, PI3K and of ERK phosphorylation has no effect on these parameters suggesting the activation of a different pathway starting at the A_2B_ receptor.

### Inhibition of the Adenosine A_2B_ Receptor Increases CD40 Expression by DC in Mice Infected by *L. amazonensis*

**Figure 5:**
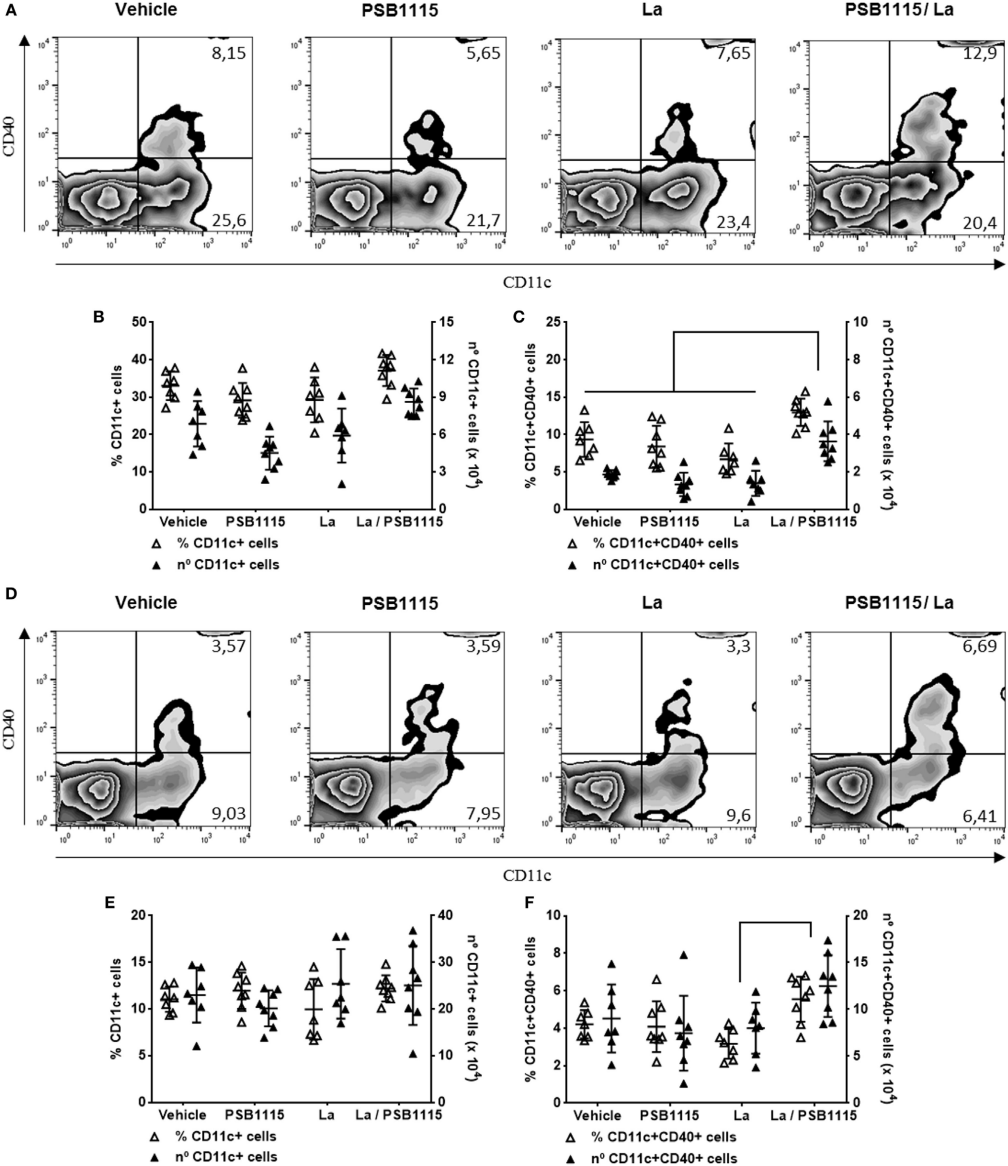
A_2B_ receptor blockade increases the percentage of CD40^+^ dendritic cells on ears and draining lymph nodes of *L. amazonensis*-infected mice. C57BL/6J mice were inoculated intradermally in both ears with 10^5^ metacyclic promastigotes of *L. amazonensis*, in the presence or absence of 5 µM PSB1115 (adenosine A_2B_ receptor antagonist). 7 days after infection, both ears and draining auricular lymph nodes were removed. The percentage or the total number of cells in both tissues of CD11c^+^ **(B,E)** or CD11c^+^CD40^+^ **(C,F)** cells in the ears **(B,C)** or lymph nodes **(E,F)** were analyzed using flow cytometry. Representative dot plots of the expression of CD11c and CD40 in the ears **(A)** or lymph nodes **(D)**. The results represent the mean + SD from two independent experiments with three or four mice per group. *p < 0.05 between linked groups, one-way ANOVA and Tukey’s post-test.

Considering that cAMP production triggered by A_2B_ receptor is important to the inhibition of CD40 in *L. amazonensis*-infected DC, that this effect is not present in infection by other species of *Leishmania* (34) and that CD40 plays a central role in the activation of T lymphocytes by DC (8), we evaluated the expression of CD40 on DC from ears and draining lymph nodes of mice infected by *L. amazonensis*, in the presence or absence PSB1115, an A_2B_ receptor antagonist. Due to its higher solubility in water, PSB1115 is more appropriate for *in vivo* studies (44) and hence was used in the following experiments. Administration of PSB1115 in the infective inoculum increases the percentage of CD40^+^ DC in both the ear and draining lymph nodes (Figure 5). The percentage of DC, evaluated by CD11c expression, on injection sites and draining lymph nodes is not modify by infection or PSB1115 treatment nor is the expression of CD40 in DC from uninfected mice (Figure 5). PSB1115 has no direct effect on the viability or proliferation of promastigotes (data not shown). These results corroborate *in vivo* our findings of the previous experiments.

### Adenosine A_2B_ Receptor Blockade Controls Lesion Development in Mice Infected by *L. amazonensis*

**Figure 6:**
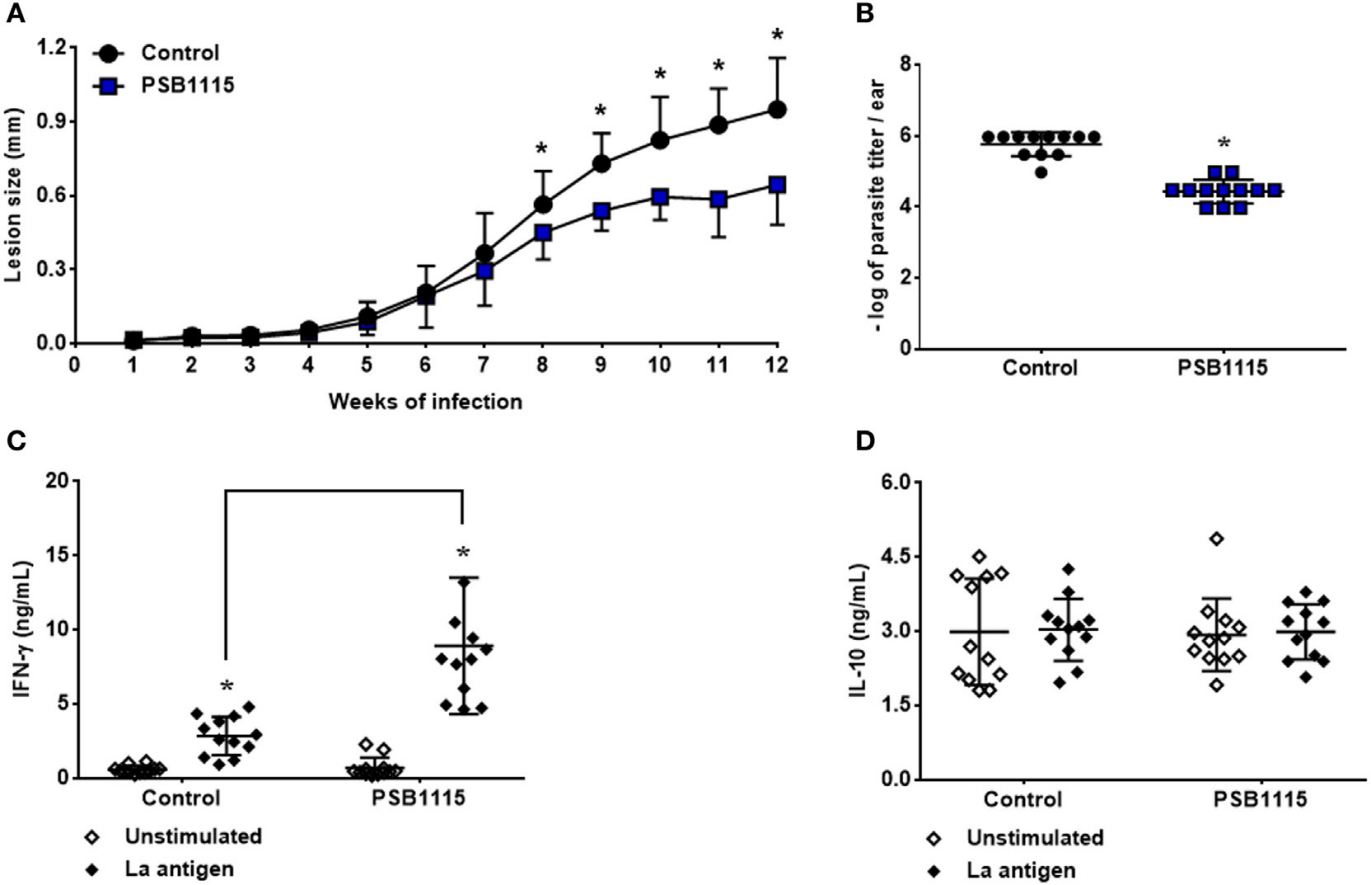
A_2B_ receptor blockade controls lesion development in mice infected by *L. amazonensis*. C57BL/6J mice were inoculated intradermally in the ears with 10^3^ metacyclic promastigotes of *L. amazonensis*, in the presence or absence of 5 µM PSB1115 (adenosine A_2B_ receptor antagonist). **(A)** Lesion sizes were measured weekly. The lesion size was defined as the difference between the infected and uninfected contralateral ear. **(B)** Tissue parasitism at the 12th week of infection. Lesions from infected mice were excised and parasitism evaluated by limiting dilution. IFN-γ **(C)** or IL-10 **(D)** production by draining lymph node cells from mice infected for 12 weeks. Cells were stimulated for 48 h with particulate antigen of *L. amazonensis*. The results represent the mean + SD from three independent experiments with four mice per group. *p < 0.05 between Control and PSB1115 groups **(A,B)** or between unstimulated and stimulated cells or linked groups **(C)**, two-tailed Student’s *t*-test **(A,B)**, two-way ANOVA and Tukey’s post-test **(C)**.

To investigate the role of the A_2B_ receptor activation on lesion development during *L. amazonensis* infection, C57BL/6J mice were inoculated in the ears with metacyclic promastigotes and lesion size measured weekly. Tissue parasitism and cytokine production were evaluated at the 12th week of infection. Interestingly, our results show that the blockade of A_2B_ receptor by PSB1115 reduces lesion size (Figure 6A) starting at the eighth week of infection. The reduction in lesion size was accompanied by a decrease in tissue parasitism (90%) after 12 weeks of infection (Figure 6B). Evaluation of cytokine production by antigen stimulated lymph node cells from treated mice demonstrated that the reduction in lesion size and tissue parasitism is associated with higher levels of IFN-γ in culture supernatants when compared with cells from control mice (Figure 6C). However, no alteration was detected on the production of IL-10 by these cells also demonstrating *in vivo* the apparent lack of role of this cytokine in *L. amazonensis* infection even with the inhibition of A_2B_ receptor at the beginning of the infection (Figure 6D).

## Discussion

*Leishmania amazonensis* infection is characterized by a deficiency in antigen-specific T cell response, which contributes to disease progression and failure in therapy (1, 2). It is, thus, relevant to study the mechanisms responsible for this anergy and DC would be a preferential target for intervention, given the essential role of these cells in the differentiation of effector T lymphocytes. Having previously demonstrated that *L. amazonensis* inhibit DC response by a mechanism dependent on A_2B_ adenosine receptor (34), here we decided to evaluate the intracellular events triggered by this receptor in infected cells.

Of the four adenosine receptors (A_1_, A_2A_, A_2B_, and A_3_), A_2A_ and A_2B_ are responsible for the main immunosuppressive effects of this nucleoside. In a previous work, we showed that A_2A_ receptor is not involved in the inhibition of *L. amazonensis*-infected DC (34); therefore, we focused this work only on the A_2B_ receptor.

The adenosine A_2B_ receptor has been shown to engage Gs or Gq proteins, capable of stimulating adenylate cyclase and phospholipase C, respectively (29). The increase in intracellular cAMP concentration by adenylate cyclase activity has been strongly associated with inhibition of immune cells, including monocytes/macrophages (32, 45), DC (40, 41, 46) and T lymphocytes (47). Here, we show, for the first time, that *L. amazonensis* infection, *via* activation of the A_2B_ receptor, increases cAMP production by DC and uses this mechanism to inhibit the co-stimulatory activity of infected cells. Furthermore, we showed that this messenger is essential for the inhibition of infected DC, since the inhibition of adenylate cyclase by SQ22536 treatment restores the ability of *L. amazonensis*-infected DC to express CD40 and produce IL-12p70.

Phospholipase C leads to the accumulation of intracellular calcium (29) that is involved in the maturation of human monocyte-derived DC (48, 49) and may be involved in the generation of a population of DC that produces low amounts of IL-12 and drives the differentiation of Th2 lymphocytes (48). Our results show that, although *L. amazonensis* infection increases the intracellular levels of calcium, this effect was independent of A_2B_ receptor triggering. In addition, NECA, a non-specific adenosine receptor agonist, did not alter the levels of calcium in infected cells, showing that although calcium may be important in *L. amazonensis*-infected DC response, the increase in intracellular calcium concentration does not seem to be dependent on the activation of adenosine receptors.

Impairment of DC activation was independent of PKA activity but triggered by a PI3K-dependent pathway. Considering that the inhibition of adenylate cyclase and the inhibition of PI3K have the same effect on DC response, and that cAMP can lead to the PI3K activation (29, 42), we suggest that the inhibition of CD40 expression and IL-12p70 production by infected DC is mediated by a cAMP-PI3K pathway.

The triggering of adenosine receptors, especially A_2B_ receptor, can lead to the activation of any of three major MAPK cascades, known as JNK, p38 and ERK1/2 (29, 30). As previously shown for macrophages infected with *Leishmania mexicana* (50), we also found that DC infection by *L. amazonensis* increases the phosphorylation of these three kinases. However, the increase of phosphorylation of JNK and p38 in infected cells was independent of A_2B_ receptor. Moreover, the inhibition of these proteins by SP600125 or SB203580 was unable to reverse the inhibition of DC activation as measured by CD40 expression and IL-12 production. It has been previously described that *L. amazonensis* stimulates the expression of phospho-ERK1/2 in infected DC (35). In another study, Schulte and Fredholm (51) demonstrated that the phosphorylation of ERK1/2 induced by the activation of the A_2B_ receptor is dependent on PI3K and independent of PKA. Our results link these previous observations by showing that ERK1/2 phosphorylation in *L. amazonensis*-infected DC is dependent on A_2B_ receptor activation and cAMP production. Our data also strongly suggest that the previously unknown G protein-coupled receptor associated with ERK1/2 phosphorylation (43) is, in fact, the adenosine A_2B_ receptor. In addition, by showing that the inhibition of CD40 expression and IL-12p70 production by DC caused by *L. amazonensis* was dependent on ERK1/2, we provide further evidence that ERK1/2 phosphorylation is associated with the pathogenesis of *L. amazonensis* infection.

Several pathogens are also able to modulate MAPK signaling (reviewed by Ref. (52)). In *Toxoplasma gondii* infection, the blockade of ERK phosphorylation decreases parasite proliferation (53) and increase IL-12 production by host cells (54), corroborating our results with *L. amazonensis* infection. Interestingly, *Trypanosoma cruzi*, another protozoan parasite, triggers ERK phosphorylation which stimulates the production of IL-10 and decreases lymphocyte proliferation by regulatory DC (55). Also, ERK phosphorylation is associated to cardiac damage induced by TGF-β in *T. cruzi*-infected mice (56). Thus, ERK phosphorylation seems to be a common observation in situations where immune modulation by pathogens occurs. However, the participation of purinergic signaling in these settings has not yet been addressed. It would be interesting to investigate the participation of adenosine mediated ERK phosphorylation in infections by these parasites.

Differently from the infection with other *Leishmania* species (57–59) IL-10 does not seem to play a relevant role in *L. amazonensis* infection (20, 60). As shown before by our group (34) and in the present study, IL-10 production by DC was not altered by *L. amazonensis* infection or by any of the treatments used. These findings contrast with the observation that ERK1/2 phosphorylation is important for IL-10 production by *L. amazonensis*-infected macrophages (36). Possible explanations for the discrepancy observed are the cell type (macrophages versus DC), the parasite stage (amastigotes versus metacyclic promastigotes), the strain of mice used (BALB/c—highly susceptible to *Leishmania* infection versus C57BL/6—resistant to most *Leishmania* species) and the fact that low molecular weight hyaluronic acid was necessary for IL-10 production. In our studies, cells were not further stimulated (present work) or were stimulated with LPS (34). Interestingly, however, the study by Yang and colleagues (36) shows that treatment of infected mice with U0126, to inhibit ERK1/2 phosphorylation, restrains parasite growth and lesion development. The finding that cAMP is involved in the regulation of DC activation is relevant in the context of *L. amazonensis* infection since it provides an explanation for the lack of role of IL-10 in this infection.

Our results confirm, to some extent, a very recent work published with human macrophages demonstrating an association between A_2B_ receptor activation and ERK1/2 phosphorylation (61). However, contrary to our results, the study reports an association between IL-10 production and A_2B_ receptor activation, although this observation was not directly tested. We believe the discrepancy between the two studies could be related to the model used and/or the high concentration of MRS1754 used in the human study which may have an overlapping action on other adenosine receptors. The inhibition of other adenosine receptors, particularly, the A_2A_ may interfere with IL-10 production Nevertheless, the study by Vijayamahantesh and colleagues reinforces our observation and extends it to the human system, thus proving the validity of our findings.

**Figure 7:**
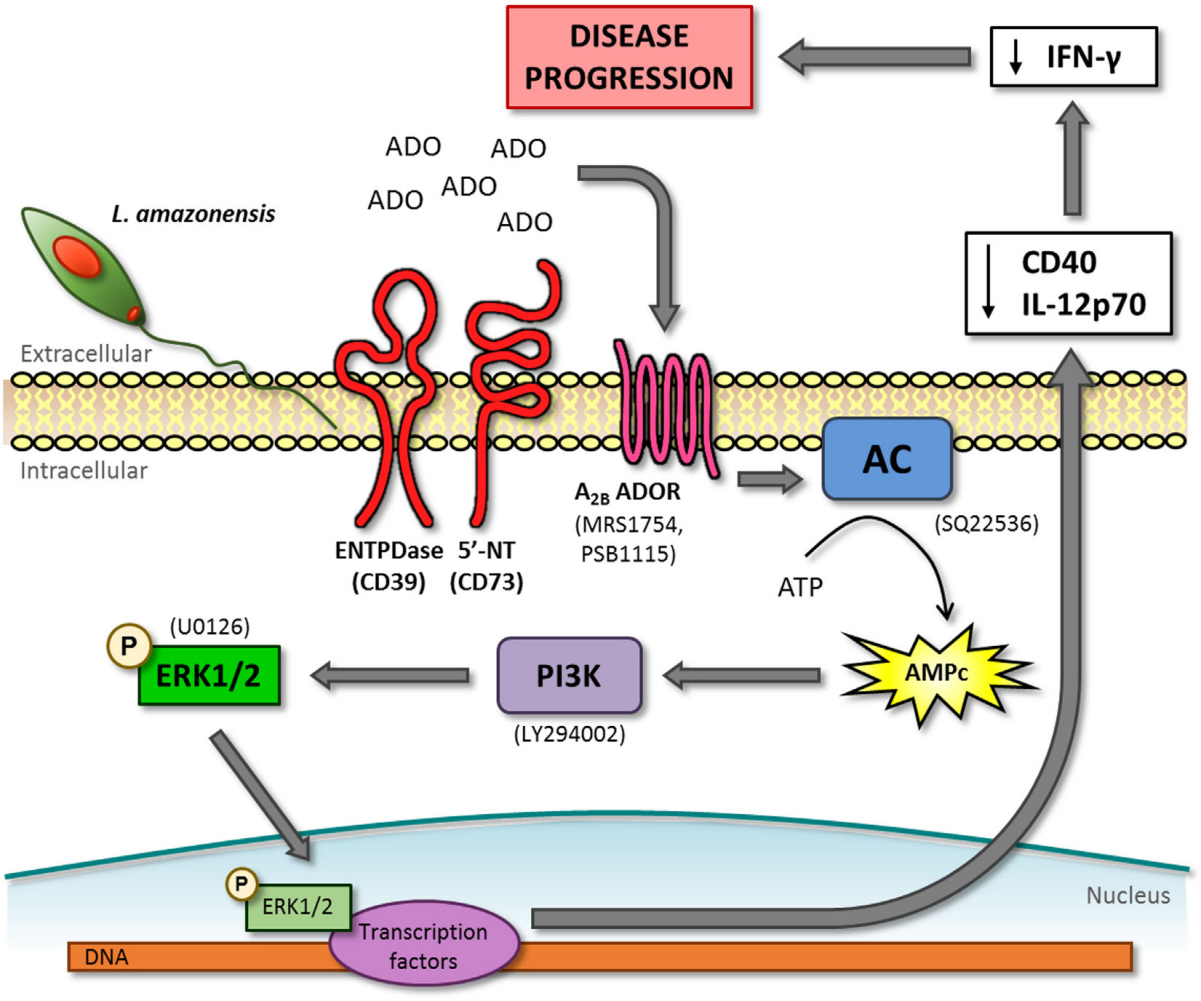
cAMP-phosphoinositide 3-kinase (PI3K)–extracellular signal-regulated protein kinases 1/2 (ERK1/2) pathway activated by the adenosine A_2B_ receptor is important to dendritic cell inhibition and lesion development in mice infected by *L. amazonensis*. *L. amazonensis* infection leads to the accumulation of adenosine (ADO) in the extracellular environment that activates adenosine A_2B_ receptor (A_2B_ ADOR) on dendritic cells (DC). The following steps of this pathway are the activation of adenylate cyclase (AC) and consequent production of cAMP, activation of PI3K and, finally, the phosphorylation of ERK1/2. In order to identify this pathway, we used inhibitors that are showed in parenthesis. phospho-ERK1/2 is able to translocate to the nucleus to interacts with transcription factors that leads to the decrease of CD40 expression and IL-12p70 production. DC inhibited by *L. amazonensis* can decrease IFN-γ production by lymph node cells, resulting in the suppression of immune response and lesion development in mice.

The results described in this report link several previous “unrelated” observations regarding the mechanism by which *L. amazonensis* inhibits the establishment of an adequate immune response. Our data implicate the activation of the adenosine A_2B_ receptor during the infection by this parasite species to the inhibition of CD40 expression (14, 35), the lack of IL-12 production (16, 35), the phosphorylation of ERK1/2 (35, 36) (Figure 7) and also provide an explanation (increased cAMP) for the inhibition of the immune response by this parasite in the absence of a Th2 response (3) and IL-10 production (20, 60). The impairment of CD40 expression and IL-12p70 production by DC caused by *L. amazonensis* is an important immune response evasion mechanism, since both molecules are essential for the development of a Th1 response necessary to control the parasite infection (7, 10). Moreover, the lower expression of these molecules could explain the lack of T cell activation observed in patients with diffuse cutaneous leishmaniasis caused by *L. amazonensis* infection (62, 63).

Corroborating this hypothesis, we demonstrated, *in vivo*, that inhibition of the A_2B_ receptor at the moment of infection not only increases CD40 expression by DC present not only in the injection site but also at the draining lymph nodes but also increases the *Leishmania*-specific Th1 response resulting in decreased lesion size and tissue parasitism. Furthermore, this enhanced Th1 response was not associated with changes in the production of IL-10 reinforcing the apparent lack of role of this cytokine in the control of the infection by *L. amazonensis* in C57BL/6 mice. Our data point to a new mechanism of control of immune response by the parasite that is associated with autocrine production of cAMP by the infected cell rather than the secretion of immunomodulatory cytokines. The fact that the treatment used in this study is not able to completely control parasite development indicates that other factors may control the enhanced Th1 response. The role of purinergic signaling on macrophages and other cells involved in the immune response against *L. amazonensis* is currently under investigation.

Finally, the recent advances of the role of purinergic signaling in the establishment and control of the immune response has triggered a series of clinical studies designed to evaluate the use of agonists, as well as antagonists, of purine receptors in different diseases with emphasis in cancer treatment (64). The confirmation of the pathway used by the *L. amazonensis* to suppress the immune response (A_2B_ receptor → cAMP → PI3K → ERK1/2) in humans may suggest possible targets for new therapeutic approaches to control *L. amazonensis* infection specially in the case of diffuse cutaneous leishmaniasis which is, as mentioned earlier, usually refractory to treatment.

## Ethics Statement

This study was carried out in accordance with the recommendations of the Brazilian Guidelines for animal experimentation. The protocols were approved by the University’s Ethical Committee on Animal Experimentation (CEUA 2012/02 and CEUA 2013/51).

## Author Contributions

Conceived and designed the experiments: AF, TM, and LA. Performed the experiments: AF and MS-T. Analyzed the data: AF, MS-T, TM, and LA. Wrote the paper: AF and LA. All authors reviewed the results and the manuscript and approved the final version of the manuscript.

## Conflict of Interest Statement

The authors declare that the research was conducted in the absence of any commercial or financial relationships that could be construed as a potential conflict of interest. The reviewers, RS and AF, and handling editor declared their shared affiliations, and the handling editor states that the process nevertheless met the standards of a fair and objective review.

## Abbreviations

DC, dendritic cell; ERK1/2, extracellular signal-regulated protein kinases 1/2; JNK, c-Jun N-terminal kinase; La, *Leishmania amazonensis*; MAPK, mitogen-activated protein kinase; NECA, 5’-(N-ethylcarboxamido) adenosine; PI3K, phosphoinositide 3-kinase; PKA, protein kinase A; PKC, protein kinase C; SAPK/p38, stress-activated protein kinases/p38.
