# Supplementary material for: Leishmania amazonensis-Induced cAMP Triggered by Adenosine A2B Receptor Is Important to Inhibit Dendritic Cell Activation and Evade Immune Response in Infected Mice
Source: Front Immunol. 2017 Jul 25;8:849. doi: 10.3389/fimmu.2017.00849 (PMC5524897; doi:10.3389/fimmu.2017.00849)
Supplement: Supplementary file 1 [file presentation_1.pdf]

Supplementary Material

Leishmania amazonensis-induced cAMP triggered by adenosine A_{2B} receptor is important to inhibit dendritic cell activation and evade immune response in infected mice

Amanda Braga Figueiredo[1], Míriam Conceição Souza-Testasicca[1,2], Tiago Wilson Patriarca Mineo[3], Luís Carlos Crocco Afonso[1,*]

[1] Laboratório de Imunoparasitologia, ICEB / NUPEB, Universidade Federal de Ouro Preto. Ouro Preto, Brazil.

[2] Coordenadoria da Área de Ciências Biológicas, Instituto Federal de Minas Gerais, Campus Ouro Preto. Ouro Preto, Brazil.

[3] Laboratório de Imunoparasitologia "Dr. Mario Endsfeldz Camargo", ICBIM, Universidade Federal de Uberlândia. Uberlândia, Brazil.

*** Correspondence:** Dr. Luís Carlos Crocco Afonso. Address: Laboratório de Imunoparasitologia, Departamento de Ciências Biológicas, ICEB / NUPEB, Universidade Federal de Ouro Preto, Campus do Morro do Cruzeiro, 35400-000, Ouro Preto, Minas Gerais, Brazil. Phone number: +55 31 3559 1701. Fax Number: +55 31 3559 1680. E-mail address:afonso@nupeb.ufop.br.

Supplementary Material and Methods

Intracellular calcium measurement

Cells were washed twice with Ca^{2+}- and Mg^{2+}-free HBSS, pH 7.4 and incubated with 5 µM Oregon Green 488® BABTA-1 (Molecular Probes, Eugene, OR, EUA) at room temperature for 1 h in the dark. Cells were washed twice in Ca^{2+}- and Mg^{2+}-free HBSS, pH 7.4, resuspended in RPMI-1640 supplemented with 10% FCS, 2 mM L-glutamine, 100 U/mL penicillin G potassium, and 50 µM β-mercaptoetanol, pH 7.2 and left 30 min at room temperature. Metacyclic promastigotes and DC were co-incubated as already described, at 33°C / 5% CO_2 for 15 min and subsequently analyzed using BD FACSCalibur™ flow cytometer. The baseline fluorescence was measured for 1 min at 33°C, cells were stimulated with 1 µM 5'-(N-ethylcarboxamido) adenosine (NECA, Sigma-Aldrich) and fluorescence immediately measured for 3 min at 33°C, then cells were stimulated with 1 µg/mL ionomycin and fluorescence immediately measured for more 2 min at 33°C. Cell acquisition was performed using BD CellQuest™ Pro software. Data analysis was performed using FlowJo software (Tree Star, Ashland, OR, USA).

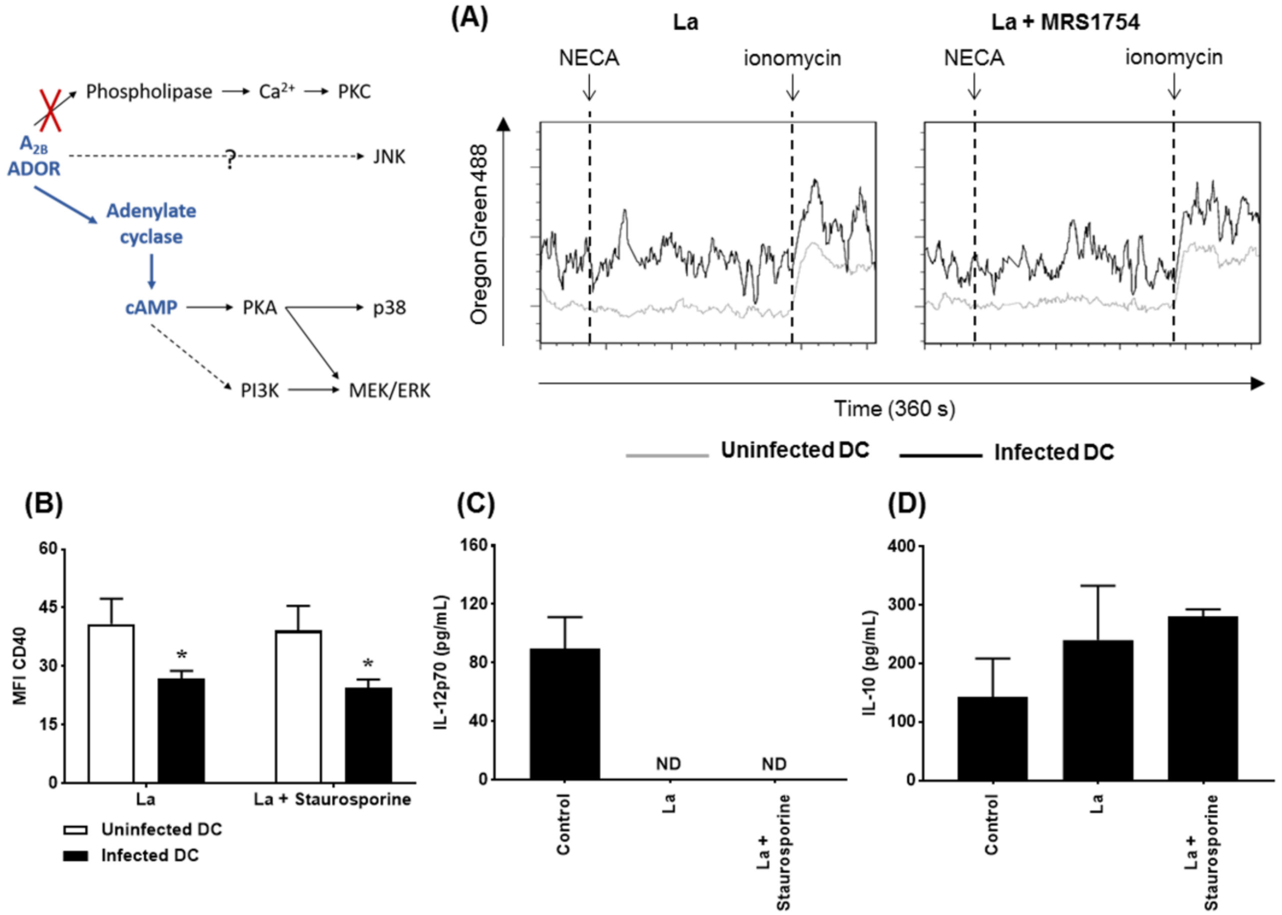

Supplementary Figure 1. ***L. amazonensis*** **infection stimulates intracellular calcium accumulation by DC by mechanisms independent on adenosine A_{2B} receptor.** (A) DC obtained after 9 days of culture with GM-CSF were infected with metacyclic promastigotes (1:3 cell to parasite ratio). (A) DC were loaded with Oregon Green 488 as described in Materials and methods and infected with PKH26-labelled *L. amazonensis* metacyclic promastigotes, in the presence of 5 µM MRS1754 (adenosine A_{2B} receptor antagonist). After 30 min of infection, we evaluated the alterations of the mean of Oregon Green 488 fluorescence prior to and following addiction of 1 µM NECA (non-selective adenosine receptor agonist) and 1 µg/mL ionomycin. DC were gated into populations of uninfected ($PKH26^-$ cells, gray line) and infected ($PKH26^+$ cells, black line) cells and the intracelular calcium accumulation monitored in both populations. Graphs are representative of two independent experiments. (B to D) DC were infected in the presence of 5 µM MRS or 1 µM Staurosporine (PKC inhibitor – Sigma-Aldrich) and CD40 expression and IL-12p70 and IL-10 production evaluated after 20 h. (B) $CD11c^+$ DC were gated into populations of uninfected ($CFSE^-$ cells, white bars) and infected ($CFSE^+$ cells, black bars) cells and the MFI of CD40 analyzed in both populations. IL-12p70 (C) and IL-10 (D) cytokine levels were measured in the supernatants using an ELISA. Control is uninfected DC. ND: not detected. The results represent the mean + SD from three independent experiments. * $p < 0.05$ between uninfected and infected DC, two-way ANOVA and Tukey's post-test.

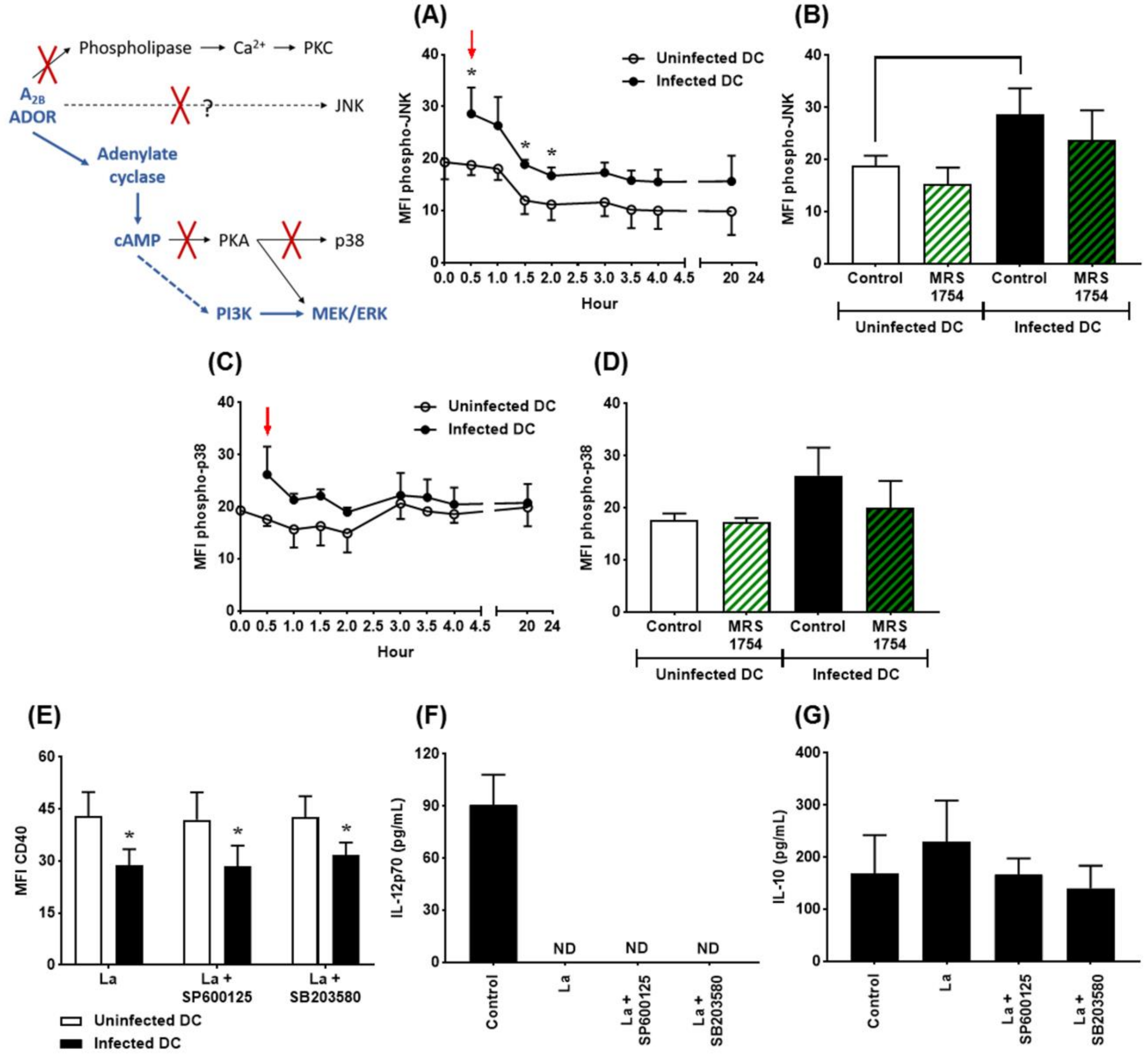

Supplementary Figure 2. JNK and p38 are not involved in the decrease of CD40 expression and IL-12p70 production by *L. amazonensis*-infected DC. DC were infected as described in figure S1 and the MFI of phospho-JNK (A) or phospho-p38 (C) analyzed in populations of uninfected and infected DC after multiple periods of infection. After infection, cells were stimulated with 1 µM NECA for 15 minutes, fixed and then analyzed by flow cytometry. MFI of phospho-JNK (B) or phospho-p38 (D) in uninfected and infected DC in the absence (Control) or presence of 5 µM MRS1754 (adenosine A_{2B} receptor antagonist), after 30 min of infection (as shown by arrow in graphs A and C). Antibodies anti-JNK pT183/pY185 (N9-66 clone) and anti-p38 MAPK pT180/pY182 (36/p38 clone) were purchased from BD Phosflow. The results represent the mean + SD from three independent experiments. * $p < 0.05$ between uninfected and infected DC or between linked groups, two-way ANOVA and Tukey's post-test. (E to G) DC were infected in the presence of 5 µM MRS1754 or 50 µM SP600125 (JNK inhibitor – Sigma-Aldrich) or 10 µM SB203580 (p38 inhibitor – Tocris Bioscience) and CD40 expression and IL-12p70 and IL-10 production evaluated after 20 h. (E) $CD11c^{+}$ DC were gated into populations of uninfected ($CFSE^{-}$ cells, white bars) and infected ($CFSE^{+}$ cells,

black bars) cells and the MFI of CD40 analyzed in both populations. IL-12p70 (F) and IL-10 (G) cytokine levels were measured in the supernatants using an ELISA. Control is uninfected DC. ND: not detected. The results represent the mean + SD from four independent experiments. * $p < 0.05$ between uninfected and infected DC, two-way ANOVA and Tukey's post-test.

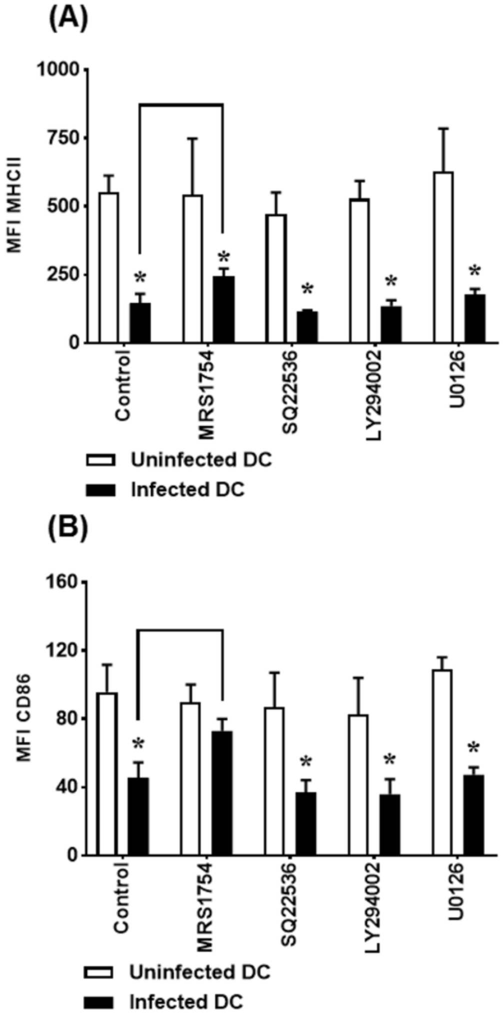

Supplementary Figure 3. Decrease of MHCII and CD86 expression in *L. amazonensis*-infected DC is independent on AMPc production, PI3K activation and ERK1/2 phosphorilation. DC were infected as described in figure S1, in the absence (Control) or presence of 5 µM MRS1754 (adenosine A_{2B} receptor antagonist), 100 µM SQ22536 (adenylate cyclase inhibitor), 1 µM LY294002 (PI3K inhibitor) or 10 µM U0126 (MEK inhibitor). After 20 h of infection, $CD11c^+$ DC were gated into populations of uninfected ($CFSE^-$ cells, white bars) and infected ($CFSE^+$ cells, black bars) cells and the MFI of MHCII (A) or CD86 (B) analyzed in both populations. Antibodies anti-mouse MHCII (M5 114.15.2 clone) and anti-mouse CD86 (GL1 clone) were purchased from eBioscience. The results represent the mean + SD from three independent experiments. * $p < 0.05$ between uninfected and infected DC or between linked groups, two-way ANOVA and Tukey's post-test.
